# Therapists’ and Researchers’ Perspectives on a Transdiagnostic Ecological Momentary Intervention “EmoGuia” for Emotional Disorders: A Consensual Qualitative Study

**DOI:** 10.3390/bs16091546

**Published:** 2026-09-01

**Authors:** Patricia Gual-Montolio, Carlos Suso-Ribera, Laura Diaz-Sanahuja, Azucena García-Palacios, Juana Bretón-López, Iratxe Alonso-Olea

**Affiliations:** 1 Department of Basic and Clinical Psychology and Psychobiology, Jaume I University, Avda. Vicent Sos Baynat s/n, 12071 Castellon de la Plana, Spain; gualp@uji.es (P.G.-M.); azucena@uji.es (A.G.-P.); breton@uji.es (J.B.-L.); 2 Faculty of Health Sciences, Universidad Europea de Canarias, 38300 La Orotava, Spain; carlos.suso@universidadeuropea.es; 3 Rheumatology Research Group, Vall d’Hebron Research Institute, 08035 Barcelona, Spain; 4 Faculty of Psychology and Speech Therapy, Universidad de Valencia, 46010 Valencia, Spain; laura.diaz-sanahuja@uv.es; 5 The Spanish Biomedical Research Centre in Physiopathology of Obesity and Nutrition (CIBERObn), Instituto de Salud Carlos III, 28029 Madrid, Spain

**Keywords:** mHealth, emotional disorders, ecological momentary assessments, transdiagnostic approach, qualitative research

## Abstract

Mental health professionals’ perspectives on transdiagnostic mobile interventions for Emotional Disorders (EDs) remain underexplored, despite their potential role in integrating ecological interventions into psychological care. This study examined the usability, acceptability, perceived clinical utility, and implementation potential of EmoGuia, a smartphone-based Ecological Momentary Intervention grounded in the Unified Protocol. Using the Consensual Qualitative Research framework, focus groups involving nine therapists and researchers were conducted following a one-week app trial. Thematic findings identified 13 categories across three domains: (1) target population, (2) satisfaction and app experience, and (3) perceived utility. Usability was additionally assessed via the System Usability Scale (SUS). Participants perceived EmoGuia as intuitive, simple, and suitable for young adults with mild-to-moderate anxiety or depressive symptoms. It was perceived as useful for fostering emotional awareness, reinforcing therapeutic content, and preventing dropout. Recommended improvements included personalized feedback, simplified language, and better notification management. SUS scores indicated high perceived usability (*M* = 79.4; SD = 12.1). These findings support EmoGuia as a promising low-intensity, adjunctive tool for early intervention in EDs and provide implementation-relevant guidance for its iterative refinement. Patient-inclusive longitudinal studies are needed to establish acceptability, engagement, feasibility, and clinical effectiveness.

## 1. Introduction

According to the latest World Health Organization (WHO) estimates, more than one billion people globally (approximately 14% of the population) live with a mental disorder (WHO, 2019, 2025). Anxiety disorders (4.4%) and depressive disorders (4.0%), collectively referred to as emotional disorders (EDs), are among the most prevalent mental health conditions worldwide and together account for more than two-thirds of all mental disorders (WHO, 2025). In Spain, prevalence rates exceed global averages, with approximately 5.2% of adults diagnosed with anxiety disorder and 5.3% with depressive disorder (Instituto Nacional de Estadística [INE], 2020; Vieta et al., 2021). This relatively high prevalence has been associated with socioeconomic disadvantages, particularly unemployment and lower social class, together with demographic factors such as gender and age (Arias-de la Torre et al., 2018). Despite their high prevalence and associated societal and economic burden, access to evidence-based mental health services remains clearly insufficient. With most countries allocating less than 2% of their healthcare budgets to mental health, there is an urgent need for increased funding to develop innovative, scalable, and effective psychological assessment and intervention protocols for individuals with EDs (Colombo et al., 2018; Hazo et al., 2017; Rathod et al., 2017).

### 1.1. Transdiagnostic Approaches and Digital Mental Health Interventions

Transdiagnostic approaches have emerged as a promising response to this challenge (Brown & Barlow, 2009; Dalgleish et al., 2020). Rather than focusing on disorder-specific symptoms, these interventions target shared emotional and cognitive mechanisms underlying EDs, thereby improving cost-effectiveness, flexibility, and personalization (Brown & Barlow, 2009; Newby et al., 2015; Pearl & Norton, 2017). Among them, the Unified Protocol (UP) stands out as the most empirically supported transdiagnostic cognitive-behavioral therapy (CBT) framework, effectively reducing anxiety, depression, and comorbid symptoms across diverse populations and treatment settings (Barlow et al., 2011, 2017). In Spain, implementations of the UP have shown strong feasibility and cost-effectiveness within the public health system (Osma et al., 2015, 2018; Peris-Baquero et al., 2022, 2023), reinforcing its value as a scalable and adaptable clinical model.

Simultaneously, Information and Communication Technologies (ICTs) have transformed the delivery of psychological interventions (Bidargaddi et al., 2020; Luxton et al., 2011). In Spain, more than 88% of the population owns a smartphone, providing an ideal platform for delivering accessible, evidence-based psychological interventions in real-world settings (Ramón-Arbués et al., 2025). Although problematic smartphone use has increased worldwide, particularly among young adults and university students (Lu et al., 2024; Ramón-Arbués et al., 2025), these technological advances have also facilitated the development of innovative assessment and intervention approaches.

### 1.2. Ecological Momentary Assessment and Intervention

Among these innovations, Ecological Momentary Assessment (EMA) enables the repeated assessment of individuals’ emotions, cognitions, behaviors, and contextual experiences in real time and within their natural environments, thereby reducing retrospective recall bias while increasing ecological validity (Gual-Montolio et al., 2022; Hallgren et al., 2017). Historically, EMA was implemented through paper-and-pencil diaries, telephone interviews, or handheld electronic devices. However, EMA has evolved considerably with the widespread adoption of smartphones, which enable automated data collection, passive sensing through embedded sensors (e.g., accelerometers and GPS), and real-time transmission of information to clinicians and researchers (Balaskas et al., 2021; Firth et al., 2017; Hallgren et al., 2017). Building upon EMA, Ecological Momentary Interventions (EMIs) extend assessment into intervention by delivering therapeutic strategies within individuals’ daily environments when they are most needed, thereby facilitating the application and generalization of psychological skills to real-world contexts (Heron & Smyth, 2010). Smartphone-based EMIs can provide personalized, context-aware, and adaptive support through just-in-time adaptive interventions (JITAIs), increasing accessibility, engagement, and adherence while reducing patient and clinician burden (Nahum-Shani et al., 2018). Recent reviews have demonstrated the effectiveness of EMI-based interventions grounded in CBT and transdiagnostic principles across a variety of populations and disorders (Daemen et al., 2021; Reininghaus et al., 2023, 2024; Schick et al., 2021), including mood and anxiety disorders (Bakker et al., 2018; Bush et al., 2014; Martínez-García et al., 2025; Schueller et al., 2017), emotion dysregulation (Castilla et al., 2022; Osma et al., 2022), gambling (Hawker et al., 2021), and severe mental illness (Lecomte et al., 2020; Linardon et al., 2024, 2025).

Despite their considerable potential, smartphone-based mental health interventions also raise important ethical considerations. Because these technologies can collect sensitive behavioral, emotional, contextual, and geolocation data, researchers and developers must ensure robust safeguards regarding privacy, confidentiality, data security, informed consent, and transparent data governance (González et al., 2024; Martinez-Martin et al., 2021). These ethical challenges become particularly relevant when EMA is employed, as it may unintentionally capture highly sensitive information about individuals’ daily routines and behaviors (Hoelscher et al., 2025). Accordingly, the implementation of digital mental health interventions should follow privacy-by-design principles and appropriate data governance frameworks to ensure the responsible use of sensitive information while fostering users’ trust and engagement (González et al., 2024).

Addressing these ethical and implementation challenges requires not only robust technological solutions but also the active involvement of the professionals who will ultimately develop, evaluate, and integrate these interventions into routine clinical practice. However, most digital mental health tools are validated primarily with patients, while the perspectives of the professionals responsible for their implementation—therapists and researchers—are rarely examined (Dao et al., 2021). Given the increasing proliferation of mental health applications (apps), these perspectives are essential for understanding apps’ usability, acceptability, and ethical soundness (Osma et al., 2022; Torous & Roberts, 2017). While therapists contribute with practical insights into feasibility and integration within real-world care, researchers provide complementary views on both the theoretical alignment and the technical feasibility (Alqahtani & Orji, 2020; Mohr et al., 2017; van Ballegooijen et al., 2014). Therefore, integrating these viewpoints is key to the sustainable adoption of EMI-based technologies in clinical settings. Additionally, this is particularly relevant given that the evaluative process of digital health technologies ideally relies on a tripartite collaboration between researchers, clinicians, and end-users, as conceptualized within user-centered design (UCD) or participatory design (PD) (Dekker & Williams, 2017).

### 1.3. Objectives of the Present Study

To address this gap, the present study explores therapists’ and researchers’ experiences using EmoGuia, a transdiagnostic smartphone app designed to deliver personalized EMIs based on users’ real-time emotional states (Gual-Montolio et al., 2023). Grounded in Barlow’s Unified Protocol (2011), EmoGuia aims to provide flexible, scalable, and evidence-based psychological support through ecological assessment and intervention. Through a one-week usability trial, this study examines professionals’ perceptions of its usability, utility, and ethical considerations. By integrating their feedback, it contributes to the limited but expanding literature on digital transdiagnostic interventions.

Therefore, the objectives of this study were (1) to explore therapists’ and researchers’ perceptions regarding the usability, acceptability, and perceived clinical utility of EmoGuia; (2) to identify strengths and areas for improvement in the app; and (3) to generate recommendations to inform its future refinement and implementation in routine clinical practice. Preliminary findings suggest generally positive perceptions regarding EmoGuia’s usability and potential clinical applicability, while also identifying important ethical and implementation challenges that should inform the future refinement and integration of digital mental health tools, such as EmoGuia, into routine care.

## 2. Materials and Methods

### 2.1. Study Design

This study employed an exploratory qualitative design to explore therapists’ and researchers’ perspectives after a one-week trial of the EmoGuia mobile app. The study followed the Consensual Qualitative Research (CQR) (Hill et al., 1997, 2005; Hill, 2012), a rigorous qualitative methodology specifically developed to investigate participants’ subjective experiences while promoting methodological transparency and analytic credibility. CQR is particularly appropriate for exploratory studies seeking an in-depth understanding of relatively underexplored phenomena because it combines the inductive principles of grounded theory (Charmaz, 2014; Glaser & Strauss, 1967) with systematic procedures designed to enhance trustworthiness through researcher consensus, reflexivity, and external auditing.

Consistent with its constructivist, phenomenological, and postpositivist foundations (Hill et al., 2005; Hill, 2012), CQR acknowledges multiple interpretations of participants’ experiences while using collaborative consensus and external review to minimize the influence of individual researcher bias. Accordingly, qualitative data were collected through semi-structured focus groups and analyzed inductively by identifying domains, constructing core ideas, and conducting a cross-analysis to derive categories that reflected common patterns across participants.

To ensure methodological rigor and reporting quality, the study adhered to the Consolidated Criteria for Reporting Qualitative Research (COREQ; Tong et al., 2007) guidelines (see Supplementary File S1). In addition, quantitative data were administered to contextualize participants’ qualitative perceptions of the app. Consistent with the exploratory purpose of the study, these quantitative data were not intended to test hypotheses or support inferential statistical analyses; they were used solely to contextualize participants’ perceptions.

### 2.2. Participants and Recruitment

Eligible participants were licensed psychologists and psychology researchers specializing in digital mental health, each with a minimum of two years of experience in either (a) the clinical treatment of patients with EDs or (b) the development or application of psychological ICTs. Therefore, some participants were eligible based on experience in one field (clinical care) while having little or no experience in the other (digital development), and vice versa, ensuring a range of perspectives from both clinical and research settings.

Recruitment followed a convenience sampling strategy through email invitations and professional networks. Colleagues and prior collaborators were invited to participate or nominate others. Although this sampling strategy may limit representativeness, it is widely used in exploratory qualitative research to recruit information from participants with relevant expertise and was considered appropriate given the specialized profile required for this study.

Sample size was determined a priori following recommendations for CQR and focus group methodology, which suggest that approximately 5–12 participants are generally sufficient to generate rich, in-depth data while maintaining manageable group dynamics and facilitating consensus-based analysis (Hill et al., 1997; Krueger & Casey, 2008). Recruitment concluded once the predefined target sample size, established according to CQR and focus group methodological recommendations, had been reached, ensuring representation of both clinicians and researchers with complementary expertise.

All participants provided written informed consent prior to participation (see Supplementary File S2), including permission for audio recording of the focus groups (see Supplementary File S3). No personally identifiable information was collected, and all data was anonymized prior to analysis to ensure confidentiality. Ethical approval was obtained from the Universitat Jaume I Ethics Committee (CD/111/2021).

### 2.3. EmoGuia App

EmoGuia—registered as “My EMI, Emotional Well-Being”—is a mobile app designed to integrate EMAs and EMIs to support self-applied, transdiagnostic interventions for individuals with EDs (Gual-Montolio et al., 2023). The app delivers brief, multimedia-based psychological exercises (images, infographics, and videos) derived from Barlow’s UP framework (Barlow et al., 2011), which are tailored to users’ daily emotional states and to key mechanisms of psychological change. While happiness was initially included as a target emotion, in this study it was replaced by guilt and shame, alongside anxiety, sadness and anger. As a result, no positive emotional state was addressed, which represents an area for improvement in future developments of the app. The mechanisms of psychological change include a more general mechanism of activity engagement, as well as core therapeutic mechanisms such as understanding the role of emotions, mindfulness, cognitive flexibility, tolerance of distress, and situational exposure (Sauer-Zavala et al., 2021).

Users complete daily EMAs at a fixed time (7:00 p.m.) to assess UP mechanisms of change, with reminders to ensure adherence. Scores below a predefined threshold for two consecutive days trigger automated alarms, prompting low-intensity EMIs. Additionally, on-demand EMAs allow users to self-report outcome variables (emotional states) at any time, with high scores (above six) also triggering an alarm and prompting tailored interventions (Chan et al., 2018; Nahum-Shani et al., 2018). Missed responses are logged to maintain ecological validity. For a detailed description of the app, including the items, pre-specified clinical alarms, and corresponding EMIs, see Gual-Montolio et al. (2023) and Table 1.

A decision-support system manages the escalation of support based on users’ self-reported data. In full clinical versions, persistent high-severity scores trigger therapist contact (e.g., phone or video call; Delgadillo et al., 2022). This feature was not used in the present study, as participants were professionals simulating user scenarios. The app is available for Android and iOS, with access granted through researcher-issued codes to ensure data control. Its design aligns with stepped-care and blended-treatment models, aiming for both experimental control and real-world scalability.

### 2.4. Measures

#### 2.4.1. Qualitative Measures

Data were collected using a semi-structured focus group interview, developed ad hoc following prior digital mental health studies (Borkovec & Nau, 1972; Castilla et al., 2023) (see Supplementary File S4). The guide explored participants’ experiences and perceptions of the app across four main domains:

1. Contributions to Therapy (6 questions)—potential clinical roles in face-to-face or blended interventions.
2. General Opinion of the App (9 questions)—usability, satisfaction, visual design, and potential improvements.
3. Use of Mobile Platforms (1 question)—advantages and limitations of smartphone delivery.
4. Confidentiality and Privacy (3 questions)—perceptions of data protection, trust, and intrusiveness.

Additionally, participants evaluated the utility and satisfaction of EmoGuia’s core tools (daily and on-demand EMAs, multimedia content, and sections for reviewing completed content) using 0–10 rating scales and open-ended questions. The interview captured both qualitative narratives and quantitative ratings, providing a multidimensional view of usability and clinical relevance.

#### 2.4.2. Quantitative Measure

The System Usability Scale—Short Form (SUS-short; Brooke, 1996; Castilla et al., 2023) was used to complement qualitative findings (see Supplementary File S5). This five-item instrument (1 = strongly disagree; 5 = strongly agree) yields a 0–100 usability score with benchmarks (Bangor et al., 2008):

- 85–100 = Best Imaginable;
- 73–84 = Excellent;
- 52–72 = Good.

The short version has shown satisfactory internal consistency (α = 0.77; Castilla et al., 2023).

### 2.5. Procedure

Participants independently installed EmoGuia and were instructed to interact with the app throughout a one-week testing period while simulating mild-to-moderate emotional symptoms. During this period, they were asked to interact with all core functionalities of the app, including completing scheduled EMAs, initiating on-demand assessments, and reviewing multimedia psychoeducational content. This testing procedure was designed to enable participants to systematically evaluate the app’s usability, perceived clinical utility, and potential integration into routine clinical practice from their professional perspective, rather than from the perspective of end users.

The focus group protocol was collaboratively developed by the research team (P.G., L.D., I.A.) and independently reviewed by the external auditor (J.B.) in line with CQR procedures (Hill et al., 1997; Hill, 2012). Before data collection, the research team discussed their professional backgrounds, prior assumptions, and expectations regarding digital psychological interventions to increase awareness of potential interpretative biases throughout the analytic process. These reflections were revisited during coding and consensus meetings to promote reflexivity in accordance with CQR recommendations (Hill et al., 2005; Hill, 2012).

Prior to conducting the focus groups, participants completed a brief sociodemographic questionnaire and provided written informed consent through Qualtrics XM (Qualtrics, Provo, UT), a secure web-based survey platform widely used in health research. Focus group sessions were subsequently scheduled using Doodle, an online scheduling platform that facilitates meeting coordination (Doodle AG, 2025).

Two virtual focus groups involving three to six participants each were conducted via Google Meet and lasted approximately 60 min. Virtual focus groups were selected because they reduce geographical and logistical barriers, facilitate participation among busy professionals, and enable the inclusion of participants from different locations without compromising interaction quality. Evidence suggests that videoconferencing platforms provide a realistic alternative to face-to-face focus groups, preserving both verbal and non-verbal communication cues while generating levels of discussion and data richness comparable to in-person groups (Kite & Phongsavan, 2017). Moreover, online videoconferencing has been shown to be a feasible, acceptable, and cost-effective method for qualitative health research, offering additional advantages such as ease of participation, secure data management, and flexibility for participants with demanding professional schedules (Archibald et al., 2019). To further promote balanced participation, focus groups were intentionally kept small (3–6 participants), allowing the moderator to encourage contributions from all participants and minimizing the likelihood that individual members would dominate the discussion (Katz-Buonincontro, 2022). All sessions were moderated by the first author (P.G.), an experienced psychologist trained in qualitative research methods. Before each session, participants were reminded of the study objectives, confidentiality procedures, and their right to withdraw at any time, and oral consent for audio recording was reconfirmed. To minimize response bias, participants were encouraged to openly discuss both positive and negative aspects of the app. The moderator followed the semi-structured interview, used neutral prompts when clarification was required, and avoided evaluative or leading questions. No detailed information about the expected outcomes of the study or hypotheses was provided in advance to preserve the spontaneity and authenticity of participants’ contributions.

All focus groups were audio-recorded, anonymized, and transcribed verbatim by four independent assistants who were not involved in the study design, data collection, or analysis. All identifying information was removed from the transcripts, and each participant received a unique identification code to preserve confidentiality. The anonymized transcripts were then analyzed by the research team following the CQR procedure described below.

### 2.6. Data Analysis

Quantitative data, including participants’ SUS scores and demographic variables, were analyzed using descriptive statistics in SPSS v23.0 (IBM Corp, 2015, Released).

For qualitative data, analysis followed the CQR framework (Hill et al., 1997, 2005) through two main stages:

1. Team Formation and Coding Preparation

The primary analysis team comprised three coders (two PhD students, P.G. and I.A., and one PhD psychologist, L.D.), all women with complementary expertise in clinical psychology, digital mental health, and qualitative research. Although the moderator (P.G.) had interacted with participants during the focus groups, the remaining analysts had no prior contact with participants, thereby reducing the potential influence of pre-existing relationships and minimizing bias on data interpretation. Consistent with CQR recommendations (Hill et al., 2005), the multidisciplinary composition of the team facilitated the consideration of multiple perspectives during data interpretation while reducing the likelihood that a single theoretical orientation would dominate the analysis. Additionally, an experienced qualitative and clinical researcher (J.B.), a woman associate professor who was not involved in data collection or coding, served as the external auditor. Following CQR recommendations (Hill et al., 2005; Hill, 2012), the auditor independently reviewed the evolving domain structure, core ideas, categories, and supporting quotations at multiple stages of the analysis, challenging interpretations, identifying inconsistencies, and ensuring that the final coding framework remained grounded in the original data.

2. Domain and Category Development

Analyses were conducted inductively using ATLAS.ti (version 24.1.1.30813; ATLAS.ti Scientific Software Development GmbH (2023), Berlin, Germany) and followed the iterative consensus process recommended in CQR (Hill et al., 1997, 2005; Hill, 2012). First, each coder independently read the transcripts several times to achieve data familiarization before identifying preliminary domains, core ideas, and categories. Subsequently, the research team then met repeatedly to compare interpretations, refine domain boundaries, and reach consensus through discussion and continual reference to the original transcripts rather than through voting or majority decisions. Disagreements were resolved through discussion until unanimous agreement was reached, with the auditor providing critical feedback whenever necessary and ensuring alignment with CQR standards.

Following CQR procedures, domains represented broad topic areas; core ideas captured the essence of each participant’s statements within these domains; and categories summarized a cross-analysis that describes consistencies in the core ideas across different domains (Hill et al., 1997, 2005; Hill, 2012). The prevalence of each category was determined according to the number of participants who contributed data to that category and was classified using the corresponding CQR frequency labels (general, typical, variant, and rare). To provide additional descriptive information regarding the relative salience of each topic within the dataset, we also report the number of coded excerpts assigned to each category. These reference counts reflect the frequency with which a topic emerged across the focus groups and are presented solely as descriptive indicators; they should not be interpreted as reflecting the number of participants endorsing a category or its relative importance.

The final structure emerged through an iterative process of constant comparison across transcripts and continuous reference to raw data to ensure validity. Throughout the analytic process, reflexivity was actively maintained by encouraging team members to critically examine their own assumptions, consider alternative interpretations, and justify coding decisions with direct reference to participants’ accounts. Consensus meetings emphasized equal participation, open discussion, and mutual respect among researchers to minimize individual bias and enhance analytic credibility. The external auditor independently reviewed each stage of the analysis, providing critical feedback that prompted further refinement whenever necessary. These procedures are central to CQR and contribute to the transparency, credibility, dependability, confirmability, and trustworthiness of qualitative findings (Hill et al., 2005).

## 3. Results

### 3.1. Participants’ Characteristics

Of the eleven professionals initially invited, nine completed the one-week trial of EmoGuia, while the remaining two were unable to attend due to scheduling incompatibility. The final sample comprised six women and three men, aged 29 to 52 years (M = 37.9, SD = 6.8). All were licensed psychologists, and several also held academic or research positions. Clinical experience ranged from 0 to 12 years (M = 7.67, SD = 3.33), while experience with ICT tools in mental health ranged from 0 to 13 years (M = 5.56, SD = 4.55).

Participants varied in their balance of clinical and technological expertise—some specialized in direct patient care, while others focused on digital intervention design. This heterogeneity was intentional, allowing for a multidimensional evaluation of the app’s usability and clinical potential. Detailed sociodemographic and professional information is presented in Table 2.

### 3.2. Qualitative Findings

Following the CQR framework, three major domains, thirteen categories, and their corresponding core ideas were identified through inductive analysis and team consensus, later reviewed by the external auditor. These domains captured (1) perceptions of EmoGuia’s target population, (2) overall satisfaction and usability, and (3) perceived utility for patients and therapists. Given the sample size (*n* = 9), frequency labels were assigned following CQR recommendations. Categories endorsed by eight or nine participants were classified as general, those represented by five to seven participants as typical, those represented by two to four participants as variant, and categories identified in only one participant as rare (Hill et al., 2005; Hill, 2012). A detailed matrix of categories, core ideas, frequency and illustrative quotes is provided in Appendix A (Table A1).

#### 3.2.1. EmoGuia’s Target Population

This domain captures participants’ views on the type of users for whom EmoGuia is most suitable. This domain was divided into four categories, ranked by the frequency of their mention:

- Type of symptomatology and severity: Participants viewed EmoGuia as most appropriate for mild or subclinical anxiety and depressive symptoms, emphasizing its preventive and adjunctive potential. For instance, one participant noted, “For mild symptoms, it can indeed be a great help” (Participant 4, P4). They agreed that individuals with severe depression or low motivation might struggle to sustain engagement, given the self-monitoring demands of the app: “With mild symptoms, because if it’s severe, you do need to have a more daily follow-up” (P3). A third participant highlighted, “In severe patients, especially those who are depressed, any small extra task requires a great effort from them” (P1).
- Motivation and commitment: This category emerged as crucial moderators of app adherence. Participants suggested that individuals who are reluctant to monitor their symptoms through other means may similarly experience difficulties engaging with the app. One participant mentioned, “In some cases, when we find that there is no motivation to record on paper, we also find that there is no commitment to using the application either” (P2). Other participants remarked, “It requires a lot of willpower to respond, to answer at the moment” (P3), and “Above all, it is necessary to have commitment” (P2).
- Age: Participants identified younger age groups, particularly those in their twenties, as more likely to benefit from the app. For example, one participant suggested, “The younger patients, I think, would be more familiar with it, particularly those in their twenties. Some even spontaneously use applications to practice more mindfulness or listen to podcasts” (P3).
- Technological resources: This category highlighted the importance of technological familiarity and resources for effective app use. Participants noted potential barriers for individuals lacking experience with technology. For example, one participant remarked, “A population that has technological resources and is generally familiar with reading and writing through their phone” (P3).

#### 3.2.2. Satisfaction, Usability and App Experience

This domain explores participants’ opinions and satisfaction regarding different features of the app. Overall, satisfaction with EmoGuia was high, with participants describing it as intuitive, simple, and visually engaging. These positive qualitative impressions were complemented by the descriptive System Usability Scale (SUS-short form) results. Following the one-week evaluation, SUS scores ranged from 55 to 95, with a mean of 79.4 (*SD* = 12.1), indicating good-to-excellent perceived usability according to established interpretative guidelines (Bangor et al., 2008). Two participants provided the highest observed score (95), whereas one participant rated usability at 55. Six of the nine participants provided scores between 75 and 85. Because the SUS was administered solely to contextualize the qualitative findings, these scores were interpreted descriptively and were not used to test hypotheses or infer clinical effectiveness.

Table 3 summarizes the SUS-short results. These quantitative findings align with participants’ qualitative feedback, underscoring EmoGuia’s intuitive design, accessibility, and clinical potential while highlighting opportunities to enhance personalization and user engagement.

Several categories were identified:

- Audiovisual psychological content: This category is related to the effectiveness of images, infographics and videos in delivering psychological transdiagnostic content. Additionally, some future proposals were suggested. Participants recommended adding reinforcement elements or self-reflection tools (e.g., notes or audio recordings). Overall, participants consistently perceived videos as more engaging and emotionally expressive than written text or static graphics: “Regarding the content, I really liked the infographics, but I think videos would be more powerful for the patients” (P3). Another participant emphasized, “The two materials that are recorded with image and audio, from my point of view, they are much more targeted, much more engaging, hearing the other person’s voice, the intonation they speak with, […]. I think it’s more impactful” (P3). Participants also proposed enhancements such as providing a space for users to write or record audio notes for specific moments. Some examples of future proposals are “A blank space where they can write or record an audio note to be more specific about what happened at that moment or something like that, right?” (P9), and “I miss a little bit of what Participant 6 mentioned about reinforcement. I think throughout the whole app, it lacks a little face that reinforces me, that tells me ‘Very well’, that it’s good that you now feel more capable of facing this, something like that” (P8).
- Psychological content adjustment to assessment responses: This category assessed how well the app’s content aligns with evaluation responses, including user alarms and notifications. Participant 3 (P3) mentioned: “I actually think that whenever you score below a specific point for some content, it should always provide you with something”. Participant 8 (P8) stated “I think the sequence is coherent”.
- Intrusiveness: This category refers to the feeling of discomfort or insecurity due to threats to confidentiality, data protection, privacy, and the perceived burden of daily notifications. Participants mentioned that perceptions of the app’s intrusiveness are subjective and depend on the users. Examples include: “For the patient who uses it, it wouldn’t be intrusive. I think those who find it intrusive are the ones who won’t engage or respond” (P3), and “There may be people who may feel the app prompts overwhelming, one more task within daily routines. But it’s true that they may express a reluctance to record on paper too” (P2).
- Opinion and future proposals of Ecological Momentary Assessment (EMA): This category refers to participants’ opinions of assessment scales and the utility of scheduled and on-demand EMAs. Participants proposed refining the response scales (e.g., Likert-type instead of numeric) and including positive emotions (e.g., joy, pride) to complement the focus on distress-related states. Some participants stated: “In line with what Participant 9 says, maybe we should rethink the response scale. I mean, instead of using a numerical scale, maybe a Likert scale could be considered, I don’t know, ranging from “not anxious” to “very anxious”. Maybe that would help to determine where to set the cutoff point” (P5), “Regarding the questions, they talk about emotions like shame, anxiety, sadness... I would add frustration, I mean, I think frustration, along with shame and guilt, is also important as a secondary emotion. Ultimately, it’s about gathering more comprehensive data” (P6), and “I don’t know if you’ve considered adding positive emotions, not just negative ones” (P5).
- Ease of use: This category refers to the app’s clean layout and straightforward navigation which facilitated engagement. Participants evaluated how user-friendly and accessible the app is in terms of its interface and overall design. Some comments were “I think it’s quite intuitive, in a rather straightforward format. I believe that for most people, they wouldn’t have a problem using it” (P4), “It’s comfortable to use, simple, and easy to understand” (P2), “Perhaps what I liked the most is the simplicity aspect; it’s very straightforward” (P5).
- Language employed: This category assesses the appropriateness and clarity of the language used in the app. A participant mentioned: “Perhaps, the language used in the videos was too technical, I don’t know... It seemed too much to me, too technical and formal, considering the type of user the app is aimed at in the end” (P5). Some professionals considered the language slightly formal or technical for end users, suggesting simplification for broader accessibility.

Overall, participants expressed confidence in EmoGuia’s usability and structure, while emphasizing refinements that could enhance personalization and emotional resonance.

#### 3.2.3. EmoGuia Perceived Utility

Participants perceived EmoGuia primarily as a complementary tool rather than a stand-alone intervention, highlighting potential benefits for both patients and therapists.

Patient-Focused Utility Subdomain:

This category underscores the app’s utility as a complementary or support tool for patients for several purposes:

- To promote emotional awareness: This subcategory refers to the potential use to enhance emotional awareness of patients through assessments. Some participants mentioned: “I become more aware of what’s happening to me, and I find solutions” (P4), “Maybe I haven’t taken a moment all day to think about how much shame, sadness, and guilt I’ve been feeling” (P4), and “It helps working on emotional awareness” (P2).
- To maintain the connection with therapy (accompaniment): This category refers to the app’s role in providing connection and accessibility to help patients to stay engaged with therapists. Some examples include: “I would give it a ten, because with the app they feel accompanied. They know that behind all this, there is someone. They have a technological tool for support, but behind it, there is a person interested in helping whom they can turn to at any time, and that is crucial” (P4).
- As a reminder: This category highlights the app’s function in providing reminders to reinforce or review therapeutic content. Participants expressed: “The app allows you to carry it with you at all times and reminds you in the moment when you need it most” (P2), “You don’t have to carry paper around or remember, there are those reminders” (P4), “The reminders help to recall what you’ve already worked on in face-to-face therapy, so you don’t forget to practice the skills you are working on” (P8).
- Dropout prevention: Participants mentioned that the app could enhance adherence to treatment, thereby preventing dropouts and, consequently, improving efficiency. Participants mentioned that: “Helping them to stay engaged and not disconnect” (P3), and “If there’s a risk for abandonment, it seems to me that it’s a way to maintain more contact with the patient on a daily basis” (P3).
- To promote behavioral change and track progress: Participants expressed “I see the application often as an anchor moment in decision-making and behavior change process” (P7), “It’s a recap of what I’ve implemented throughout the day, reflecting on things I should change for tomorrow, like taking stock of the entire day” (P4).

Therapist-Focused Utility Subdomain:

This category highlights the app’s role as a complementary tool for therapists for different purposes:

- Time reduction: This subcategory refers to the potential use of the app to reduce the time therapists spend on certain therapeutic tasks. Participants mentioned the need for an initial investment in teaching to use the app but emphasized its potential impact afterward. For example, “There needs to be an initial investment in teaching to use the app, but that can lead to saving time during therapy sessions” (P5), “It reduced time therapists need to invest” (P6), and “The records are already there, quantified, so you don’t have to go through everything, or make corrections” (P4).
- Patient monitoring: This utility for therapists focuses on the ease of monitoring patient progress. For example, some participants said that: “It will show the patient’s evolution between evaluations” (P4), “I believe it can help the therapist became aware of whether the patient is implementing strategies or not” (P2), “To continue verifying on a daily basis whether they are following or implementing the points we have worked on in their emotional psychoeducation” (P2).
- Precision of EMAs: This subcategory refers to the apps’ utility in recording daily objective assessments of patients, rather than relying on retrospective recall. Participants mentioned: “Many patients come in saying they haven’t been doing so badly. Then, as you start asking, you realize that things have been quite rough. Or sometimes it’s the opposite, where someone describes a horrible week, but daily assessments show it hasn’t been that bad, suggesting a generalization effect from a single day or event that clouds the rest of the week” (P4), “In that moment, it can provide a clearer picture of the situation” (P7), “The advantage of having the app with us in the moment for “ecological assessment” (P4), and “The questionnaire was very short and concise, and that is appreciated” (P9).

Collectively, these findings suggest that participants valued EmoGuia as a potential blended-care adjunct—a bridge between therapy sessions and real-life emotion regulation contexts.

## 4. Discussion

### 4.1. Main Findings and Perceived Clinical Applicability

The present study explored therapists’ and researchers’ perspectives following a one-week simulated evaluation of EmoGuia, a smartphone-based Ecological Momentary Intervention (EMI) grounded in Barlow’s Unified Protocol (UP) for transdiagnostic treatment of emotional disorders (EDs) (Barlow et al., 2011). Overall, therapists and researchers perceived EmoGuia as an intuitive and potentially valuable tool for supporting psychological treatment, while also identifying opportunities to improve personalization, engagement, and accessibility. As one of the first qualitative studies to specifically explore professionals’ perspectives on an EMA- and EMI-based smartphone intervention grounded in the UP, the present findings contribute to the growing literature on ecological transdiagnostic interventions and provide valuable guidance for the iterative refinement of digital psychological tools before large-scale clinical evaluation.

The qualitative analysis yielded three overarching domains: Target Population, Satisfaction and app Experience, and Perceived Utility, providing a comprehensive understanding of professionals’ perceptions regarding the strengths, limitations, and future development of EmoGuia. Overall, professionals generally perceived the app as most suitable for individuals experiencing mild-to-moderate emotional symptoms, particularly those who were motivated to engage in self-monitoring and comfortable using digital technologies. These perceptions are consistent with previous evidence indicating that low-intensity digital psychological interventions are particularly appropriate for individuals with subclinical or less severe symptomatology, whereas patients with more severe emotional disorders often require more intensive therapist support (Hetrick et al., 2018).

Accordingly, participants viewed EmoGuia primarily as a complement to therapist-guided treatment rather than a stand-alone intervention. They highlighted its potential to enhance emotional awareness, reinforce therapeutic learning between sessions, and promote the transfer of emotion regulation skills to daily life. These observations are consistent with previous research on digital implementations of the UP (Celleri et al., 2025; Martínez-García et al., 2025) and further support the integration of ecological smartphone interventions within blended-care and stepped-care models (Bakker et al., 2016, 2018; Castilla et al., 2022; Linardon et al., 2025). Additionally, participants perceived the app as a useful mechanism for maintaining a sense of therapeutic continuity between sessions and potentially reducing dropout, a recurrent challenge in both traditional and digital interventions (Torous et al., 2020). These findings extend those reported on the RegulEm app (Osma et al., 2022; Martínez-García et al., 2025), in which usability and clinical integration were also identified as key implementation factors. Unlike previous smartphone adaptations of the UP that mainly reproduce therapeutic modules within mobile platforms, EmoGuia combines EMA with personalized EMIs delivered in real time according to users’ emotional states, representing an ecological extension of the UP into everyday life.

Quantitative usability findings were consistent with these qualitative perceptions. The System Usability Scale (SUS-short form) score (M = 79.4) indicates good-to-excellent usability (Bangor et al., 2008) and is comparable to previous evaluations of UP-based mobile interventions (Martínez-García et al., 2025). Although these findings do not demonstrate clinical effectiveness, they support the feasibility of future implementation studies.

Despite these encouraging findings, professionals identified several areas for improvement. Professionals consistently emphasized that sustained engagement with digital mental health interventions depends not only on usability, but also on personalization, motivational support, digital literacy, and minimizing assessment burden (Osma et al., 2022; Robillard et al., 2019; Torous et al., 2018). Their recommendations—including adaptive notifications, richer multimedia resources (e.g., positive reinforcement or gamified feedback), simplified language, positive emotion tracking, and more individualized feedback—closely align with current evidence on JITAIs and digital mental health interventions (Nahum-Shani et al., 2018; Delgadillo et al., 2022). These recommendations are particularly relevant considering a recent meta-analysis showing that transdiagnostic smartphone interventions produce small but significant improvements in depression, anxiety, and psychological distress compared with control conditions (Linardon et al., 2025). However, the review also highlighted considerable variability in engagement and relatively high attrition rates, identifying sustained user engagement as one of the principal challenges facing digital mental health interventions.

### 4.2. Implementation and Ethical Considerations

The present findings also help clarify the complementary roles of internet-based interventions and smartphone apps within digital mental healthcare. Although both approaches aim to increase access to evidence-based psychological treatments, they differ substantially in their mode of delivery (Celleri et al., 2025; Delgadillo et al., 2022). Internet-based interventions usually deliver structured therapeutic modules, whereas ecological smartphone interventions provide continuous monitoring and context-sensitive support throughout daily life (Nahum-Shani et al., 2018). Rather than competing approaches, both formats may play complementary roles within blended-care models, with web-based programs delivering structured treatment and smartphone apps reinforcing therapeutic skills in real-world contexts (Celleri et al., 2025). From this perspective, EmoGuia should not be understood simply as a mobile version of the UP but as an ecological extension of its transdiagnostic principles into everyday life.

Beyond usability, the present findings also highlight important implementation challenges associated with ecological mobile interventions. Participants identified younger adults with mild-to-moderate emotional symptoms and adequate digital literacy as the population most likely to benefit from the current version of the app. However, successful engagement with EMIs appears to depend on more than digital literacy alone. Recent evidence suggests that EMIs also require sufficient cognitive bandwidth, that is, the attentional and executive resources necessary to respond to ecological prompts and initiate self-regulation in real time (Jaremba et al., 2026). During periods of acute psychological distress, elevated stress, or emotional overload, these cognitive resources may become temporarily compromised, making individuals less likely to engage with smartphone-delivered interventions precisely when support is most needed. Consequently, barriers to implementation are not solely technological but also cognitive and emotional, particularly among individuals experiencing greater symptom severity. These observations are consistent with broader concerns regarding digital exclusion in mental healthcare, whereby unequal access to technology, functional limitations, and differences in cognitive or digital competence may inadvertently widen existing health inequalities despite the potential of digital interventions to improve access to care (Beneito-Montagut et al., 2022; Martinez-Martin et al., 2021).

These findings suggest that the challenge for future ecological interventions is not only determining when support is needed but also when users have sufficient cognitive capacity to benefit from it. Consequently, future iterations of EmoGuia should move beyond generic accessibility principles and incorporate specific implementation and accessibility-oriented strategies aimed at reducing both technological and cognitive barriers to engagement. Potential adaptations include simplified interfaces requiring fewer interactions, reduced cognitive demands during periods of elevated distress, adaptive notification schedules based on users’ current emotional state, brief low-effort interventions (e.g., grounding or breathing exercises) during episodes of acute stress, postponement of cognitively demanding reflective exercises until emotional arousal decreases, optional voice-assisted interaction for older adults or users with limited digital literacy, and caregiver- or clinician-supported monitoring when clinically appropriate.

Importantly, these recommendations should be interpreted within the intended scope of EmoGuia. The app was conceived as an ecological complement to therapist-guided psychological treatment rather than as a stand-alone intervention. Its primary purpose is to reinforce the acquisition and generalization of UP skills within blended- and stepped-care models, or to provide low-intensity support for individuals presenting with subclinical or mild-to-moderate emotional symptoms. Accordingly, the accessibility adaptations proposed here should not be interpreted as strategies to replace clinician-guided care for individuals with severe or highly complex emotional disorders, but rather as approaches to broaden equitable access within the target population for which the intervention was designed.

Finally, although professionals primarily discussed issues related to notification burden and perceived intrusiveness, concerns regarding privacy, data governance, and the management of sensitive ecological data were comparatively limited. Rather than suggesting that these issues are unimportant, this finding may reflect the increasing normalization of digital data collection among professionals accustomed to implementing digital health technologies. Nevertheless, future implementation research should explicitly examine stakeholders’ perceptions of data privacy, transparency, trust, and governance alongside usability and acceptability outcomes, as these factors are likely to influence the successful and ethical implementation of ecological mental health interventions (González et al., 2024).

Overall, the findings suggest that the principal contribution of smartphone-based EMIs lies not simply in digitizing existing psychological interventions but in extending evidence-based therapeutic principles into everyday life. By combining continuous ecological assessment with personalized, context-sensitive interventions, apps such as EmoGuia have the potential to facilitate the generalization of therapeutic skills beyond therapy sessions, strengthen self-management, and reinforce continuity of psychological care. Importantly, these conclusions should be interpreted as reflecting therapists’ and researchers’ perspectives following a one-week simulated evaluation and therefore provide guidance for the refinement and future implementation of the application rather than evidence of clinical effectiveness in patient populations.

### 4.3. Strengths and Limitations

The present study has several strengths that contribute to the growing literature on digital mental health interventions. First, unlike many usability studies that focus primarily on end users, this research examined the perspectives of therapists and researchers, two stakeholder groups that play a fundamental role in the development, evaluation, and implementation of digital psychological interventions before large-scale effectiveness trials. This study is also consistent with user-centered and participatory design principles, which advocate the active involvement of multiple stakeholders throughout the development of digital health technologies (Alqahtani & Orji, 2020; Dekker & Williams, 2017). Second, the use of the CQR methodology, together with independent coding, consensus procedures, and external auditing, enhanced the credibility and trustworthiness of the qualitative analysis. The complementary descriptive SUS data further contextualized participants’ perceptions of usability. Despite these strengths, several limitations should be considered. First, the small convenience sample, recruited partly through professional networks, may have included participants with greater familiarity with digital mental health and more favorable attitudes toward digital interventions. Consequently, confirmation bias cannot be completely excluded, and the findings should be interpreted as informed professional perspectives rather than representative views of clinicians or researchers more broadly. Second, participants evaluated EmoGuia during a one-week simulated evaluation while simulating mild-to-moderate emotional symptoms. Consequently, the findings should not be interpreted as evidence of patient acceptability, engagement, or clinical effectiveness, but rather as guidance for the iterative refinement of the intervention prior to feasibility and effectiveness testing in clinical populations. Likewise, the short evaluation period may also have limited assessment of longer-term engagement, adherence, and changes in user experience. Finally, objective app usage metrics (e.g., interaction time, frequency of use, EMA completion rates, or engagement trajectories) were not collected. Consequently, it was not possible to examine the relationship between participants’ subjective evaluations and their actual patterns of interaction with the app. Future studies integrating qualitative methods with objective digital behavioral data may provide a more comprehensive understanding of engagement processes and implementation barriers.

### 4.4. Implications for Implementation and Future Research

Finally, although this study focused on professionals’ perspectives, the successful implementation of ecological mobile interventions ultimately depends on the experiences of end users. Therefore, future research should evaluate EmoGuia and other smartphone transdiagnostic interventions in more heterogeneous samples of mental health professionals with varying levels of experience in digital interventions, as well as in clinical populations through longitudinal feasibility studies and randomized controlled trials. These studies are needed not only to establish the app’s clinical effectiveness, acceptability, implementation outcomes, and cost-effectiveness across diverse healthcare settings (Dao et al., 2021), but also the mechanisms underlying sustained engagement, predictors of intervention receptivity, and the extent to which ecological interventions facilitate the transfer and generalization of therapeutic skills into everyday life.

Future research should continue to adopt user-centered design principles by actively involving patients, therapists, researchers, and other stakeholders throughout the iterative refinement process (Schaeuffele et al., 2025). Particular attention should be devoted to developing increasingly adaptive ecological interventions capable of responding to users’ momentary emotional, cognitive, and functional needs. Accordingly, future versions of EmoGuia should prioritize greater personalization, adaptive notification systems, simplified user interfaces, reduced cognitive burden during ecological assessments, voice-assisted interaction where appropriate, caregiver-supported functionalities for users requiring additional assistance, and transparent ethical data governance. Moreover, future JITAI algorithms should investigate whether tailoring the timing and complexity of ecological interventions according to users’ momentary cognitive and emotional capacity improves long-term engagement while reducing digital exclusion (Jaremba et al., 2026).

## 5. Conclusions

Overall, the present findings suggest that smartphone-based ecological transdiagnostic interventions, such as EmoGuia, may represent a promising approach to extending evidence-based psychological care beyond the therapy setting by reinforcing emotion regulation skills in everyday contexts. Although clinical effectiveness remains to be established through longitudinal feasibility studies and randomized controlled trials, the present study provides valuable evidence supporting the usability, acceptability, and implementation potential of an ecological transdiagnostic intervention from the perspective of mental health professionals following a one-week simulated evaluation of the app, thereby informing the iterative refinement of EmoGuia prior to feasibility and effectiveness testing in clinical populations. Continued stakeholder involvement, user-centered design, and rigorous ethical governance will be essential to maximize the clinical impact and scalability of future digital mental health interventions. Future development should also prioritize personalization, adaptive JITAI algorithms, and implementation strategies that optimize long-term engagement while minimizing cognitive burden and preserving equitable access across diverse user populations.

## Figures and Tables

**Table 1:** Correspondence EMA-EMI in EmoGuia.

Item	EMA	EMI
1	Sadness	Image with psychoeducation of emotion’s function Infographics to check the facts and observe the function of the emotion Video with behavioral activation based on goals and values
2	Anger	Image with psychoeducation of emotion’s function Infographics to check the facts and observe the function of the emotion, STOP exercise and problem solving Video with tools to reduce the intensity of anger
3	Anxiety	Image with psychoeducation of emotion’s function Infographics to check the facts, observe the function of the emotion and analyze the anxiety’s curve Video of a validation technique of anxiety.
4	Shame	Image with psychoeducation of emotion’s function Infographics to observe the function of the emotion and validate it Video with tools to reduce the intensity of shame and validate it
5	Guilt	Image with psychoeducation of emotion’s function Infographics to manage emotion Video with tools to reduce the intensity of guilt and validate it
6	Activity level	Image with psychoeducation of relation between activity and emotional well-being Infographics with steps for behavioral activation based on values and goals Video of behavioral activation based on values and goals.
7	Understanding the role of emotions	Image of the adaptive function of emotions Infographics with the model of the 3 components of emotions and examples of the 3 components of emotions Video of “Identifying emotions”
8	Mindfulness	Image of Acceptance of emotional experiences without judgment Infographics of Resignation vs. Acceptance Video of a Mindfulness Exercise
9	Cognitive flexibility	Image of the vicious cycle of catastrophic thoughts Infographics about Cognitive Reevaluation and Flexibility Video of how to think more flexible
10	Tolerance to unpleasant physical sensations	Image with psychoeducation of physical sensations Infographics about how to deal with uncomfortable physical sensations Video of Tolerance to unpleasant physical sensations
11	Behaviors not guided by emotion	Image of adaptive behaviors not guided by emotions Infographics of opposite action Video of opposite action

**Table 2:** Socio-demographic and professional characteristics of participants (*n* = 9).

Participant	Gender	Age	Professional Role	Clinical Experience (Years)	ICT Experience (Years)	Patients Treated
1	Woman	33	Psychologist	6	1	60a
2	Man	39	University professor	10	10	0
3	Woman	33	Psychologist	8	3	50a
4	Man	29	PhD student	0	4	0
5	Man	47	Psychologist	8	3	15/20b
6	Woman	36	Psychologist and university professor	11	13	20b
7	Woman	36	Psychologist and university professor	12	12	15b
8	Woman	36	Psychologist	8	4	20b
9	Woman	52	Psychologist	6	0	25b

Note. Clinical experience refers to years of practice with patients presenting EDs. ICT experience refers to participants’ exposure to digital tools in clinical or research contexts. Patient data refer to cumulative totals (a) or estimated number of patients per week (b), as reported by participants.

**Table 3:** Individual System Usability Scale (SUS) scores.

Participants	SUS ^1^
1	75
2	75
3	75
4	85
5	55
6	80
7	95
8	80
9	95

^1^ SUS, System Usability Scale.

## Data Availability

The original contributions presented in this study are included in the article/Supplementary Material. Further inquiries can be directed to the corresponding author.

## Supplementary Materials

The following supporting information can be downloaded at: https://www.mdpi.com/article/10.3390/bs16091546/s1, Supplementary File S1: Table S1: Consolidated Criteria for Reporting Qualitative Research (COREQ) guidelines (Tong et al., 2007); Supplementary File S2: Participants’ informed consent; Supplementary File S3: Audio recording consent form; Supplementary File S4: Semi-structured Interview for Focus Groups (in English); Supplementary File S5: Short-form of System Usability Scale.

## Appendix A

**Table A1:** Results of the content analysis of therapists’ experience focus groups.

Domains	Categories (CQR Frequency; Nº of Coded Excerpts)	Subcategory	Illustrative Core Idea
Target Population	Type of symptomatology and severity (General; *n* = 27)		This mobile app might be more appropriate for mild or subclinical anxiety and depressive symptoms. While it could also be used in more severe cases, especially depression, it will require more intensive and personalized measures, as even small tasks can be a significant effort for these patients. For patients with anxiety symptoms, the app could serve as a distraction technique.
	Motivation and Commitment (for change) (Typical; *n* = 8)		Effective use of this mobile app requires a certain level of motivation and commitment from the patient. More motivated individuals are more likely to use it effectively.
	Age (Typical; *n* = 7)		The mobile app could be suitable for young adults who are more familiar with technology, whereas its use among older adults or very young individuals may cause some discomfort.
	Technological Resources (Variant; *n* = 5)		The app is more effective for users with digital literacy and technological resources, making it easier for them to read information through their smartphones.
Satisfaction and App Experience	Audiovisual psychological content (General; *n* = 88)		There is a preference for audiovisual content in the app as it allows better understanding and accessibility. Videos and infographics are particularly clear, precise, and useful. As a future proposal, the app should be more flexible and personalized, offering additional resources such as positive reinforcement, more accessible visual content, and options for interaction and communication (e.g., audio recording), to facilitate detailed and personalized communication.
	Psychological content adjustment to assessment responses (General; *n* = 37)		Despite the clarity and usefulness of the content, some therapists desire deeper engagement through the app and greater personalization, along with adjustments in the scoring system and response times to enhance the experience and effectiveness.
	Intrusiveness (General; *n* = 21)		Some might find the app minimally intrusive due to their familiarity with notifications, while others may feel overwhelmed by them, which could negatively impact engagement and adherence. For patients with moderate depressive symptoms, the app might be perceived as an additional burden, potentially causing further distress in their daily routine.
	Opinion and future proposals of Ecological Momentary Assessment (EMA) (General; *n* = 13)		It is important to rely on validated assessment scales to establish cut-off points, categorize the content applied to subclinical or more severe populations, and consider a greater variety of emotions. It is suggested to further adjust assessment scales to improve feedback precision and utility.
	Ease of Use (Typical; *n* = 10)		The mobile app is simple, intuitive, and easy to use, making it accessible and comfortable for most users, including those with depressive symptoms.
	Language Employed (Typical; *n* = 9)		For users less familiar with psychological content, the app content may pose a barrier. Adjusting the tone of audiovisual content and technical language could enhance accessibility and clarity for users.
Perceived utility	Prevention (Typical; *n* = 7)		This app can serve as a preventive tool, offering highly appropriate psychoeducational information.
	Patient-Focused Utility (*n* = 72)	Emotional Awareness (General; 24)	The app’s evaluation questions promote reflection and enhance emotional awareness.
		Connection with therapy (General; 18)	The app connects patients with therapists throughout the day, providing support.
		Reinforcement and Reminder (General; 19)	The app could reinforce therapy by providing daily practice and frequent reminders, ensuring constant access to therapeutic content.
		Dropout Prevention (Typical; 8)	The app helps to prevent treatment dropout, and facilitates help-seeking when experiencing difficulties, especially for those prone to avoidance or with depressive symptoms.
		Promote Behavioral Change and track Progress (Variant; 3)	Through the app, patients are encouraged to change behaviors and track their progress.
	Therapist-Focused Utility (*n* = 43)	Time Reduction (General; 16)	Despite the initial investment required for therapists, the app can reduce the time spent on correcting questionnaires, monitoring records, and overall workload.
		Patient Monitoring (General; 16)	The app provides therapists with detailed information on patient progress, facilitating data collection on mood and learned skills.
		Precision of Ecological Momentary Assessment (General; 11)	The app’s real-time recording of emotions and mechanisms of change helps therapists to have more accurate and detailed evaluations, providing a realistic and specific view of emotional progress, overcoming the limitations of retrospective memory.
