# Supplementary material for: Therapists’ and Researchers’ Perspectives on a Transdiagnostic Ecological Momentary Intervention “EmoGuia” for Emotional Disorders: A Consensual Qualitative Study
Source: Behav Sci (Basel). 2026 Sep 1;16(9):1546. doi: 10.3390/bs16091546 (PMC13603596; doi:10.3390/bs16091546)
Supplement: Supplementary file 1 [file behavsci-16-01546-s001.zip › behavsci-4397710-supplementary.pdf]

Supplementary file S1.

Table S1. Consolidated Criteria for Reporting Qualitative Research (COREQ) guidelines (Tong et al., 2007).

Topic	Item No.	Guide Questions/Description	Reported on Page No.
		**Domain 1: Research team and reflexivity**	
		Personal characteristics	
Interviewer/facilitator	1	Which author/s conducted the interview or focus group?	Section 2.5
Credentials	2	What were the researcher's credentials? E.g., PhD, MD.	Section 2.6
Occupation	3	What was their occupation at the time of the study?	Section 2.6
Gender	4	Was the researcher male or female?	Section 2.6
Experience and training	5	What experience or training did the researcher have?	Section 2.6
		Relationship with participants	
Relationship established	6	Was a relationship established prior to study commencement?	Section 2.5
Participant knowledge of the interviewer	7	What did the participants know about the researcher? e.g., personal goals, reasons for doing the research.	Section 2.5
Interviewer characteristics	8	What characteristics were reported about the interviewer/facilitator? e.g., Bias, assumptions, reasons and interests in the research topic.	Section 2.6
		**Domain 2: Study design**	
		Theoretical framework	

Methodological orientation and Theory	9	What methodological orientation was stated to underpin the study? e.g., grounded theory, discourse analysis, ethnography, phenomenology, content analysis.	Section 2.1
		Participant selection	
Sampling	10	How were participants selected? e.g., purposive, convenience, consecutive, snowball.	Section 2.2
Method of approach	11	How were participants approached? e.g., face-to-face, telephone, mail.	Section 2.2
Sample size	12	How many participants were in the study?	Section 2.2, Section 3.1 & Table 2
Non-participation	13	How many people refused to participate or dropped out? Reasons?	Section 3.1
		Setting	
Setting of data collection	14	Where was the data collected? e.g., home, clinic, workplace.	Section 2.5 & Section 2.6
Presence of non-participants	15	Was anyone else present besides the participants and researchers?	NA
Description of sample	16	What are the important characteristics of the sample? e.g., demographic, data.	Section 3.1 & Table 2
		Data collection	
Interview guide	17	Were questions, prompts, guides provided by the authors? Was it pilot tested?	Section 2.4.1 & Supplementary file 4
Repeat interviews	18	Were repeat interviews carried out? If yes, how many?	Section 2.5

Audio/visual recording	19	Did the research use audio or visual recording to collect the data?	Section 2.5 & Supplementary file 3
Field notes	20	Were field notes made during and/or after the interview or focus group?	NA
Duration	21	What was the duration of the interviews or focus group?	Section 2.5
Data saturation	22	Was data saturation discussed?	NA
Transcripts returned	23	Were transcripts returned to participants for comment?	NA
		**Domain 3: analysis and findings**	
		Data analysis	
Number of data coders	24	How many data coders coded the data?	Sections 2.5 & 2.6
Description of the coding tree	25	Did authors provide a description of the coding tree?	Section 2.6
Derivation of themes	26	Were themes identified in advance or derived from the data?	Section 2.1 & Section 2.6
Software	27	What software, if applicable, was used to manage the data?	Section 2.6
Participant checking	28	Did participants provide feedback on the findings?	NA
		Reporting	
Quotations presented	29	Were participant quotations presented to illustrate the themes/findings? Was each quotation identified? e.g., participant number	Section 3.2 & Table A1
Data and findings consistent	30	Was there consistency between the data presented and the findings?	Section 4

Clarity of major themes	31	Were major themes clearly presented in the findings?	Section 3.2 & Table A1
Clarity of minor themes	32	Is there a description of diverse cases or discussion of minor themes?	Section 3.2, Table A1 & Section 4.1

Supplementary file S2. Participants' informed consent.

INFORMED CONSENT – THERAPISTS

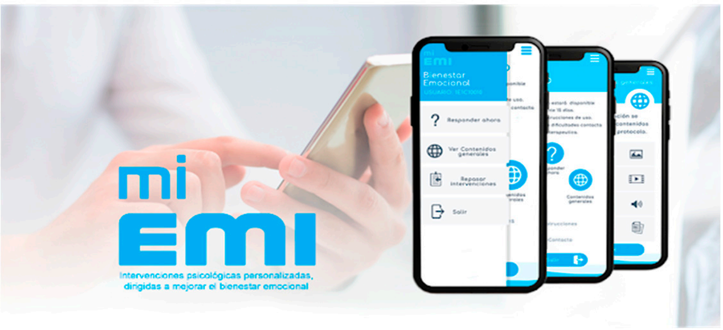

Figure S1. *Overview of the EmoGuia app interface and functionalities.*

Research Project Title: Use of Technology to Guide and Improve Psychotherapy Outcomes

Principal Investigator: Juana María Bretón López

Study Sponsors: Jaume I University

Objectives: The aim of this study is to (a) investigate the feasibility of a mobile application ("EmoGuia") to personalize psychological content and improve mood in individuals with mild anxiety and/or depression symptoms, and (b) examine user expectations, satisfaction, and usability of the app. Additionally, participation offers an opportunity to access evidence-based psychological content at no cost and to gain relevant information for managing emotions.

Procedure: Your participation as a therapist will help evaluate the clinical utility of a mobile application that monitors emotional state and delivers personalized psychological content based on responses over one week. While using the app, you will receive psychological content depending on daily assessment questions. If needed, a psychologist from the research team will assist with any questions regarding the implementation of the intervention strategies provided by the app. Upon completion of the study, a group interview will be conducted to gather your feedback and satisfaction with the app.

Benefits: There is no financial compensation for participating in this study. However, using this app may help improve your mood.

For any questions, contact Patricia Gual Montolio (gualp@uji.es).

This study has been approved by the Ethics Committee of Jaume I University (File No. CD/111/2021). Your data in this study will be kept strictly confidential for research purposes.

- I have read the study information sheet.
- I am of legal age (18 years or older).
- I understand that I may withdraw from the study:
    - At any time
    - Without providing any explanation

Supplementary file S3. Audio recording consent form.

Full Name: ______________________________

One of the resources of the Psychology and Technology Laboratory (LabPsiTec) at Jaume I University is audio recording interviews regarding the mobile app. These recordings provide highly valuable information, which will be transcribed and analyzed qualitatively for research purposes aimed at improving relevant psychological interventions.

STATEMENT:

- I have been adequately informed about the use of the audio recording of this interview conducted by the LabPsiTec research group at Jaume I University.
- I understand that my right to privacy and anonymity will always be protected and voluntarily consent to the audio recording of this interview.
- I acknowledge that, based on this voluntary consent, I may withdraw my permission and request that the recording not be conducted at any time.

Participant Signature: ______________________________

Castellón, _____ of ________________, 2024

Supplementary file S4. Semi-structured Interview for Focus Groups (in English).

(The original interview was conducted in Spanish and has been translated for publication)

Opinion Interview on the "EmoGuía" App for Therapists and Researchers

The questions below aim to gather your opinions and experiences regarding the use of the "EmoGuía" app. The goal is to understand how the learning and practice of the resources you have received during this period have benefited you, as well as the app's usefulness. We would like you to answer each question as openly and fully as possible. The information collected from this interview will be used anonymously and exclusively for research purposes.

BLOCK 0

Before continuing with the interview, please complete the following sociodemographic survey: https://jaumei.eu.qualtrics.com/jfe/form/SV_em4u2iYzu8aAg6O

BLOCK 1: Contributions to Therapy

- What do you think the use of the EmoGuía app could contribute to in-person therapy and/or blended therapy?
- For what type of population would you use this app?
- Would you use it as a standalone intervention (only the app) or as a complement to another form of therapy (face-to-face or self-applied)? Why?

- Regarding the practice of certain psychological tools and skills (mindfulness, cognitive flexibility, etc.), how does the app encourage their practice? Do you think it helps users train these skills, or not?
- Do you think the use of the app could serve as a first step that encourages or motivates people to seek more intensive or ongoing help?
- On a scale from 0 to 10 (0 = not helpful at all; 10 = indispensable), to what extent do you think the app could help prevent dropout from a treatment (self-applied or face-to-face)? Why?

BLOCK 2: General Opinion of the App

- Do you think it would be advisable for your patients to use this app? Would you recommend it to them? Would you integrate it into your clinical or research practice? Why or why not?
- What did you like most or find most interesting or engaging about using it? Why?
- What do you think about the level of complexity when using it?
- How did you find the visual design (contents)?
- What positive effects have you found in the app? What are its main benefits or advantages?
- What negative effects or potential drawbacks do you think the app might have?
- Would you like it to include any additional features it does not currently have? Which ones?
- What do you think about time efficiency for therapists? Do you think it helps reduce human and time resources, or could it require more therapist involvement?
- What do you think about the app providing psychological content based on users' daily assessment responses?

BLOCK 3: Opinion About the Mobile Format

- What advantages and disadvantages do you see in having it as a mobile phone application?

BLOCK 4: Confidentiality

- Did you feel confident using a mobile app? Do you think your patients would trust it? Why? If not, how could that be improved?
- Did you feel your privacy was invaded or interfered with?
- Was it easy for you to manage notifications without feeling uncomfortable?

Evaluation questions on Cross-Cutting tools of the app

We will now talk about specific tools or sections of the app. Please answer as openly as possible.

1. **6 Scheduled EMA (Ecological Momentary Assessment) questions at 7:00 p.m.**

These questions addressed psychological processes (behavioral activation, experiential avoidance, emotional awareness, cognitive flexibility, mindfulness, physical sensations). They consisted of a daily assessment at 7:00 or 8:00 p.m. After completing it, the app provided a resource if necessary.

The **sequence** of the scheduled processes was:

Behavioral activation → Experiential avoidance → Emotional awareness → Cognitive flexibility → Mindfulness → Physical sensations.

- How useful were these questions for you as a user of the app? (0 = not useful at all, 10 = extremely useful)
- How useful do you think these questions would be for your patients?
- What aspects of these scheduled questions did you find most satisfying?
- Would you add any other process-related questions? Which ones? Why?
- The app triggered psychological content after two consecutive days, scoring below 2 in a specific process. Do you think two days were appropriate, or would you have preferred a shorter or longer time frame? Would you make it more or less demanding?

**Usefulness**	0	1	2	3	4	5	6	7	8	9	10
**Satisfaction**	0	1	2	3	4	5	6	7	8	9	10

2. "Respond Now" Section

This section allowed you to answer five questions about your emotions when you were experiencing peak distress (anxiety, sadness, anger, shame, guilt).

- How useful were these questions for you? (0–10)
- How useful do you think they would be for your patients?
- What aspects of these on-demand questions did you find most satisfying?
- Would you add any questions about other emotions? Which ones? Why?
- What do you think about being able to respond in the moment of greatest distress?

The app could only deliver one piece of content at a time, even if two high-scoring alarms were triggered. The **order of alarms** was anxiety → sadness → anger → shame → guilt.

- What did you think about this order for delivering psychological content? Would you rearrange it or prioritize any emotion differently (e.g., sadness before anxiety or anger)?

**Usefulness**	0	1	2	3	4	5	6	7	8	9	10
**Satisfaction**	0	1	2	3	4	5	6	7	8	9	10

3. Psychological Content (Images, Infographics, and Videos)

- Which psychological content did you like the most? Any specific image, infographic, or video?
- Which psychological content do you think could help patients the most?
- Would you add any other content?
- Do you think the app provided personalized feedback/content based on your responses to the alarms? Did it match your needs?
- What do you think about receiving resources in order of increasing psychological intensity (image → infographic → videos → video call with the therapist)? Did this sequence add value to you?
- Did you feel represented in the written content (e.g., tone, gender, wording)?

- What did you think about images and videos being displayed horizontally, while infographics were shown vertically?

4. **"Review My Content" Section**

In this section, you can access all previously viewed materials to review them whenever needed.

- How useful was this section for you?
- What aspects did you find most satisfying?

**Usefulness**	0	1	2	3	4	5	6	7	8	9	10
**Satisfaction**	0	1	2	3	4	5	6	7	8	9	10

5. **"General Content" Section**

In this section, you could access general videos about the app and technique practice.

- How useful was this section for you?
- What aspects did you find most satisfying?
- What other videos or content would you include in this section?

**Usefulness**	0	1	2	3	4	5	6	7	8	9	10
**Satisfaction**	0	1	2	3	4	5	6	7	8	9	10

6. **Question on Your Ability to Manage Emotion Before and After Receiving Psychological Content (image, infographic, or video)**

- What did having this question before and after receiving the psychological content do for you? How did it help?
- What aspects of this question did you find most satisfying?

**Usefulness**	0	1	2	3	4	5	6	7	8	9	10
**Satisfaction**	0	1	2	3	4	5	6	7	8	9	10

Please rate the usefulness and satisfaction of each of the following aspects using a 0–10 scale:

**Aspect**	**Usefulness (Does it help you address your emotional response/problem?)**	**Satisfaction (Did you feel represented—quality, format?)**
Images	0–10	0–10
Infographics	0–10	0–10
Videos	0–10	0–10

Aspect	Usefulness (Does it help you address your emotional response/problem?)	Satisfaction (Did you feel represented—quality, format?)
Contact the psychologist (if needed)	0–10	0–10

Supplementary file S5. Short-form of SUS.

SUS, System Usability Scale

Spanish validation: Castilla, D., Jaén, I., Suso-Ribera, C., García-Soriano, G., Zaragoza, I., Bretón-López, J., Mira, A., Diaz-García, A., & García-Palacios, A. (2023). Psychometric Properties of the Spanish Full and Short Forms of the System Usability Scale (SUS): Detecting the Effect of Negatively Worded Items. International Journal of Human–Computer Interaction, 0(0), 1–7. https://doi.org/10.1080/10447318.2023.2209840

Please answer the following questions on a scale from 1 (Strongly disagree) to 5 (Strongly agree):

Strongly disagree Strongly agree

1	2	3	4	5

1. I think that I would like to use this system frequently.
2. I found the system unnecessarily complex.
3. I thought the system was easy to use.
4. I think that I would need the support of a technical person to use this system
5. I found the various functions in this system were well integrated
6. I thought there was too much inconsistency in this system
7. I would imagine that most people would learn to use this system very quickly
8. I found the system very cumbersome to use
9. I felt very confident using the system
10. I needed to learn a lot of things before I could get going with system.
